# Re-examining the effect of door-to-balloon delay on STEMI outcomes in the context of unmeasured confounders: a retrospective cohort study

**DOI:** 10.1038/s41598-019-56353-7

**Published:** 2019-12-27

**Authors:** Chee Yoong Foo, Nick Andrianopoulos, Angela Brennan, Andrew Ajani, Christopher M. Reid, Stephen J. Duffy, David J. Clark, Daniel D. Reidpath, Nathorn Chaiyakunapruk

**Affiliations:** 1 National Clinical Research Centre, Kuala Lumpur, Malaysia; 2 School of Pharmacy, Monash University Malaysia, Bandar Sunway, Selangor Malaysia; 3 Jeffrey Cheah School of Medicine and Health Sciences, Monash University Malaysia, Bandar Sunway, Selangor Malaysia; 4 Centre of Cardiovascular Research & Education in Therapeutics, Department of Epidemiology and Preventive Medicine, School of Public Health and Preventive Medicine, Monash University, Melbourne, VIC Australia; 5 Department of Cardiology, Royal Melbourne Hospital, Melbourne, Australia; 6 Department of Medicine, University of Melbourne, Melbourne, Australia; 7 School of Public Health, Curtin University, Perth, WA Australia; 8 Department of Cardiology, Austin Hospital, Melbourne, Australia; 9 Department of Cardiology, Alfred Hospital, Melbourne, Australia; 10 Molecular, Genetic & Population Health Sciences, University of Edinburgh, Edinburgh, UK; 11 Department of Pharmacotherapy, College of Pharmacy, The University of Utah, Salt Lake City, UT, USA

**Keywords:** Interventional cardiology, Health services

## Abstract

Literature studying the door-to-balloon time-outcome relation in coronary intervention is limited by the potential of residual biases from unobserved confounders. This study re-examines the time-outcome relation with further consideration of the unobserved factors and reports the population average effect. Adults with ST-elevation myocardial infarction admitted to one of the six registry participating hospitals in Australia were included in this study. The exposure variable was patient-level door-to-balloon time. Primary outcomes assessed included in-hospital and 30 days mortality. 4343 patients fulfilled the study criteria. 38.0% (1651) experienced a door-to-balloon delay of >90 minutes. The absolute risk differences for in-hospital and 30-day deaths between the two exposure subgroups with balanced covariates were 2.81 (95% CI 1.04, 4.58) and 3.37 (95% CI 1.49, 5.26) per 100 population. When unmeasured factors were taken into consideration, the risk difference were 20.7 (95% CI −2.6, 44.0) and 22.6 (95% CI −1.7, 47.0) per 100 population. Despite further adjustment of the observed and unobserved factors, this study suggests a directionally consistent linkage between longer door-to-balloon delay and higher risk of adverse outcomes at the population level. Greater uncertainties were observed when unmeasured factors were taken into consideration.

## Introduction

Door-to-balloon (D2B) time is an in-hospital process indicator of reperfusion timeliness in primary percutaneous coronary intervention (pPCI)^1^. The emphasis of achieving shorter D2B times in pPCI is based on the theoretical deduction of improved myocardial salvage with shorter ischemia-reperfusion interval. Backed by a body of real-world observational evidence, this idea had spurred a widespread focus of D2B time improvement across geographical regions over the past decade.

More recently, the focus for reperfusion timeliness has shifted from the “D2B” time to the broader measure of “contact-to-device” time following the 2017 European Society of Cardiology STEMI management guideline^2^. Contact-to-device time has advantages in capturing the various sub-components of reperfusion delay within the system of STEMI care. Yet, despite being a more comprehensive indicator of “system delay”, particularly in settings where prehospital system is better developed, contact-to-device time is less relevant in regions where prehospital system remains immature^3^. Hence, D2B time being a measure of the in-hospital processes, remains highly relevant under these circumstances.

Over the past decade, health systems in the developed world have reported significant improvement in the timeliness of reperfusion therapy via pPCI based on the D2B time indicator^4,5^. However, more recent evaluation of the improvements in D2B time shows a lack of population effect^4,6^. Population effect is referred as the average differences in outcomes between the exposed and unexposed across all units in a population. The findings raised questions about the causal nature of the time-outcome relation at the population level ^4,6,7^. The argument also elevated the fact that not all existing evidence are pointing at a significant effect that D2B delays has on ST-elevation myocardial infarction (STEMI) outcomes^4,7^. Recent review^8^ indicated that large proportion of the available evidence lack adequate confounder consideration. Only 20% of the reported analyses have considered some aspects of the confounding domains. Importantly, merely one^9^ (out of the 35 studies reviewed) has considered at least some aspects of all the identified confounding domains. Common confounding domains that lack consideration in many of the previous reports include pre-hospital delays, day-time and institutional factors.

In addition, existing studies reported almost exclusively the effect of D2B delay on STEMI outcomes in relative term, based mainly on conventional regression method. The population-level effects of D2B delay on STEMI outcomes in absolute term remain largely unknown^8^. Relative estimates derived using regression adjustment, particularly the logistic and proportional hazard models, have important limitations. Firstly, they do not provide the magnitude of the effect size in absolute term, which is important to population-level decision making. Secondly, an adjusted relative effect via regression (e.g. odds ratio) can only be used to infer to the “average” subpopulation specific to the particular regression model. This subpopulation is often arbitrary and is difficult to relate to in real-life^10,11^. Generalizing this quantitative relationship to the population level can be misguiding^12,13^.

Hence, in this study, we considered (1) the covariates balancing propensity score (CBPS) method to consider the accessible, observed factors captured in the study database; (2) a distance-based instrumental variable (IV) analysis to further consider the unobserved confounders. These methods allowed us to address the concerns of the observed and unobserved factors potentially biasing the time-outcome relationship. Additionally, the population-average effects of D2B delay on STEMI outcomes were also examined and reported, which provide the information important to population health decision makers. We hypothesized that longer D2B delay is related to a higher population-level risk of adverse STEMI outcomes despite the additional confounding consideration.

## Results

### Study cohort and crude comparison

**Figure 1:**
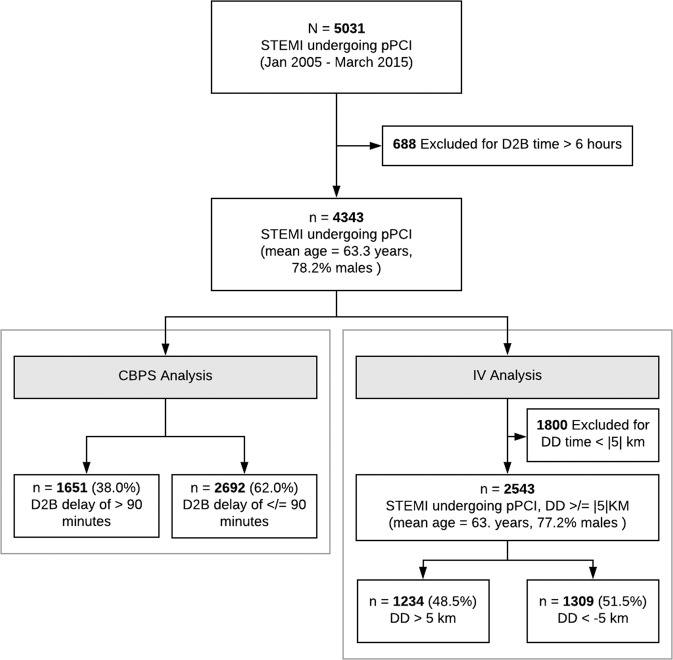
Derivation of the study cohort for propensity score analysis and instrumental variable analysis. D2B = Door-to-balloon, pPCI = Primary percutaneous coronary intervention, STEMI = ST-elevation myocardial infarction, CBPS = Covariates balancing propensity score, IV = instrumental variable, DD = Differential distance.

The MIG PCI registry included data for 5031 patients who underwent primary PCI between January 2005, and March 2015. We excluded 688 patients who had D2B times in exceeds of 6 hours. 4343 patients (mean age was 63.2 years, with 78.2% males) fulfilled the study criteria. 1651 of them (38.0%) experienced a D2B delay of >90 minutes. Figure 1 depicts further details of the number of subjects included in the CBPS analysis and IV analysis.

The cohort median D2B time was 88.5 (SD = 51.4) minutes. Median D2B time was 58.8 minutes (SD = 19.2) in the <90 minutes stratum and 136.9 minutes (SD = 50.9) for the ≥90 minutes stratum. Table 1 shows a selection of the study cohort characteristics at baseline and stratified by D2B time. Overall, those who have experienced a longer D2B delay were found to be systematically differed from those with shorter D2B time. For instance, the longer D2B time subgroup were more likely to be older, of female sex, lower smoking prevalence, more likely to have a history of heart failure within the past 2 weeks, has higher pre-procedural TIMI flow and of higher baseline Killip class. Many of these factors are indicative of a higher risk of adverse outcomes. They also differed systematically by their angiographical characteristics. Weekend admission and off-hour presentation were related to longer D2B time. (See full details in Appendix A-5) Crude event rates for all study outcomes were higher for those with longer D2B delay (Table 2).

**Table 1:** Baseline characteristics of the study cohort – overall and by D2B time experience.

*No. of patients*	Overall	D2B time ≤90 mins	D2B time >90 mins	Std. Diff
	*N* = *4343*	*n* = *2692*	*n* = *1651*	
Age, mean (sd)	63.29 (12.81)	62.79 (12.47)	64.09 (13.31)	0.101
Male, n (%)	3398 (78.2)	2152 (79.9)	1246 (75.5)	0.108
Smoking history, n (%)				0.09
Current	1545 (35.6)	988 (36.7)	557 (33.7)	
Prior	1205 (27.7)	751 (27.9)	454 (27.5)	
Never	1502 (34.6)	906 (33.7)	596 (36.1)	
Missing/Unknown	91 (2.1)	47 (1.7)	44 (2.7)	
Congestive heart failure (within 2 weeks), n (%)	259 (6.0)	130 (4.8)	129 (7.8)	0.133
Missing	2 (0.0)	0 (0.0)	2 (0.1)	
Pre-procedural TIMI flow, n (%)				0.193
0	3053 (70.3)	1976 (73.4)	1077 (65.2)	
1–2	665 (15.3)	380 (14.1)	285 (17.3)	
3	620 (14.3)	335 (12.4)	285 (17.3)	
Missing	5 (0.1)	1 (0.0)	4 (0.2)	
Killip class, n (%)				0.146
1	3160 (72.8)	2014 (74.8)	1146 (69.4)	
2	463 (10.7)	272 (10.1)	191 (11.6)	
3	100 (2.3)	48 (1.8)	52 (3.1)	
4	352 (8.1)	197 (7.3)	155 (9.4)	
Not recorded	60 (1.4)	31 (1.2)	29 (1.8)	
Missing	208 (4.8)	130 (4.8)	78 (4.7)	
Cardiogenic shock				
(Pre-procedure), n (%)	465 (10.7)	248 (9.2)	217 (13.1)	0.125
Previous MI, n (%)	553 (12.7)	305 (11.3)	248 (15.0)	0.11
Missing	9 (0.2)	5 (0.2)	4 (0.2)	
Congestive heart failure, n (%)	73 (1.7)	30 (1.1)	43 (2.6)	0.111
Missing	9 (0.2)	6 (0.2)	3 (0.2)	
Right coronary lesion, n (%)	1794 (41.3)	1233 (45.8)	561 (34.0)	0.243
Circumflex lesion, n (%)	466 (10.7)	252 (9.4)	214 (13.0)	0.115
Obtuse marginal branch lesion, n (%)	223 (5.1)	106 (3.9)	117 (7.1)	0.138
Reference vessel diameter < = 2.5 mm, n (%)	837 (19.3)	430 (16.0)	407 (24.7)	0.217
Number of stents in procedure, n (%)				0.141
1	280 (6.4)	144 (5.3)	136 (8.2)	
2	3161 (72.8)	2001 (74.3)	1160 (70.3)	
3	737 (17.0)	435 (16.2)	302 (18.3)	
4 or more	165 (3.8)	112 (4.2)	53 (3.2)	
Weekend admission, n (%)	1222 (28.1)	664 (24.7)	558 (33.8)	0.202
Off-hour presentation, n (%)	2075 (47.8)	1120 (41.6)	955 (57.8)	0.333

TIMI = Thrombolysis in Myocardial Infarction; MI = Myocardial infarction; sd = standard deviation, Std. Diff = standardized difference.

**Table 2:** Crude and adjusted relative and absolute effect of D2B delay on STEMI outcomes.

*Comparison by D2B time*	D2B time		Relative Risk (95% CI)		Risk Difference (95% CI)	
	≤90 mins	>90 mins				
No. of patients	n = 2692	n = 1651				
**Primary outcomes**			***Unadjusted***	p-value	***Unadjusted***	p-value
In-hospital mortality, n (%)	134 (5.0)	167 (10.1)	2.03 (1.63, 2.53)	<0.001	5.14 (3.47, 6.81)	<0.001
30-Day mortality, n (%)	145 (5.4)	183 (11.1)	2.06 (1.67, 2.54)	<0.001	5.70 (3.96, 7.44)	<0.001
**Secondary outcomes**						
In-hospital arrhythmia, n (%)	488 (18.1)	367 (22.2)	1.23 (1.09, 1.38)	<0.001	4.10 (1.62, 6.58)	<0.001
In-hospital shock, n (%)	188 (7.0)	188 (11.4)	1.63 (1.34, 1.98)	<0.001	4.4 (2.59, 6.21)	<0.001
In-hospital MACE, n (%)	574 (21.3)	452 (27.4)	1.28 (1.15, 1.43)	<0.001	6.05 (3.41, 8.70)	<0.001
30-Day MACE, n (%)	888 (33.0)	649 (39.3)	1.19 (1.10, 1.29)	<0.001	6.32 (3.37, 9.27)	<0.001
**Primary outcomes**			***CBPS adjusted***		***CBPS adjusted***	
In-hospital mortality	—	—	1.52 (1.15, 1.88)	0.001	2.81 (1.04, 4.58)	0.002
30-Day mortality	—	—	1.58 (1.21, 1.95)	<0.001	3.37 (1.49, 5.26)	<0.001
**Secondary outcomes**						
In-hospital arrhythmia	—	—	1.13 (0.97, 1.29)	0.090	2.78 (−0.49, 6.02)	0.092
In-hospital shock	—	—	1.25 (0.99, 1.51)	0.034	1.93 (0.05, 3.80)	0.044
In-hospital MACE	—	—	1.15 (1.01, 1.28)	0.029	3.25 (0.23, 6.27)	0.034
30-Day MACE	—	—	1.09 (0.98, 1.20)	0.080	3.22 (−0.26, 6.71)	0.069
***Comparison by differential distance***	**Differential distance**					
	**<−5** **km**	**>5** **km**				
No. of patients*	n = 1309	n = 1234				
**Primary outcomes**					***2SLS IV adjusted***	
In-hospital mortality, n (%)	77 (5.9)	96 (7.8)	—	—	20.7 (−2.6, 44.0)	0.081
30-Day mortality, n (%)	83 (6.3)	104 (8.4)	—	—	22.6 (−1.7, 47.0)	0.068
**Secondary outcomes**						
In-hospital arrhythmia, n (%)	220 (16.8)	247 (20.0)	—	—	31.3 (−5.3, 67.9)	0.094
In-hospital shock, n (%)	91 (7.0)	110 (8.9)	—	—	23.1 (−1.5, 47.7)	0.066
In-hospital MACE, n (%)	266 (20.3)	297 (24.1)	—	—	34.5 (−4.6, 73.7)	0.084
30-Day MACE, n (%)	415 (31.7)	437 (35.4)	—	—	37.4 (−7.0, 81.7)	0.099

D2B = Door-to-balloon, STEMI = ST-elevation myocardial infarction, MACE = Major adverse cardiac event, CBPS = Covariates balancing propensity score, 2SLS = two stage least square, IV = instrumental variable.

### Effect estimates by CBPS analysis

**Figure 2:**
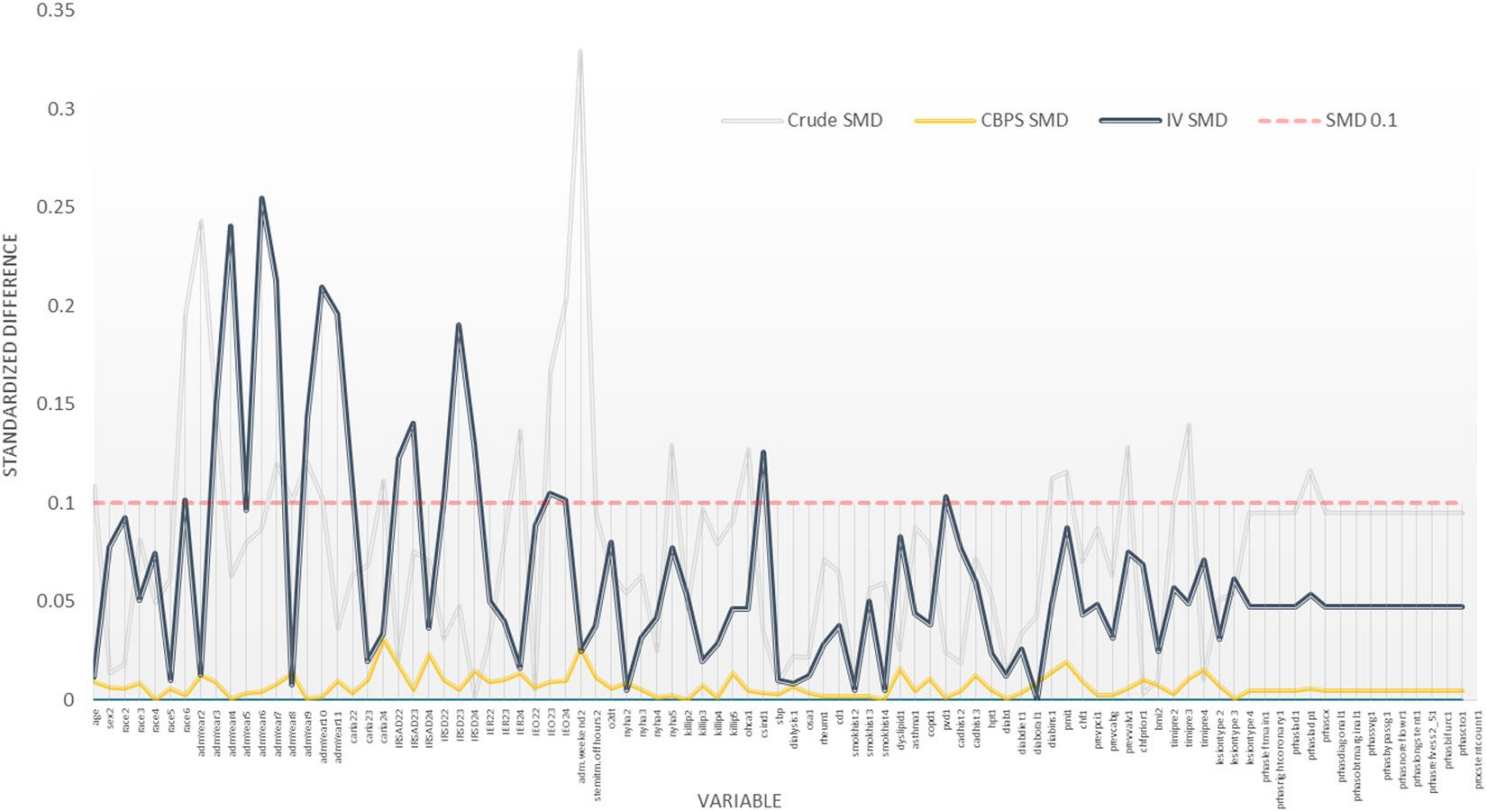
Balance of observed factors in between the comparison groups. SMD = Standardized mean difference, CBPS = Covariates balancing propensity score, IV = Instrumental variable.

Application of the CBPS model weights substantially improved the covariate balance between the comparator groups (Fig. 2 & Appendixs A-6 and A-7). All variables included in the CBPS model show no observable differences (all SMD < 0.1). The CBPS adjusted effect estimates for all study outcomes and their corresponding bootstrapped confidence interval are presented in Table 2. Overall, longer D2B delay, after CBPS adjustment, remained significantly associated with an increased risk of in-hospital and 30-day mortality as well as in-hospital shock and MACE. Notably, the magnitude of the effect sizes has reduced in comparison to the crude rates. The effect estimates for in-hospital arrhythmia and 30-day MACE has marginally exceeded the pre-determined alpha (0.05) after CBPS weighting.

### Effect estimates by IV analysis

2543 patients remained for the IV analysis after the excluding those resided in areas of DD < 5 kilometers. (Fig. 1) Among them, 1234 (48.5%) patients had a DD of >5 km. The partial F statistics estimated in the first stage of the 2SLS was 36.6, which indicates that the IV performs well in predicting the exposure variable (i.e. D2B <90 and ≥90), hence can be considered an instrument with adequate strength. DD also appears to be a reasonably valid instrument as illustrated by an improved balance of the observed characteristics in between the instrument strata (Fig. 2). Most observed factors achieved adequate balance with the SMD of <0.1. A particular concern of using DD as an instrument in this case was the potential of it being related to pre-hospital delay. If DD is related to pre-hospital delay, there is a possibility that DD may affect patient’s outcome independently of D2B time, thereby rendering this instrument invalid (i.e. violation of the IV assumption). Our assessment however, indicates the absence of a statistical relationship between DD and pre-hospital delay (represented by the onset-to-door time (o2dt) variable in the dataset, see Appendix A-6). Other observed factors that have remained imbalance included the Index of Cardiac Accessibility and Remoteness, Index of Relative Socio-economic Disadvantage, Index of Economic Resources, Index of Education and Occupation, cohort year and systolic blood pressure (SBP) (See Appendixs A-6 and A-7). These imbalances indicate that the instrument is likely to be systematically associated with patient’s socioeconomic status and treatment year. To account for these observed differences, we included these variables into the 2SLS model. For SBP, we observed that despite a marginally higher SMD for SBP between the two exposure subgroups, the absolute mean difference of SBP between the two subgroups was small (only 3.36 mmHg). This was judged to be clinically non-significant. Along with the generally well-balanced covariates of similar nature (e.g. Killip’s class) which represented adverse outcome risks, we believe that this small imbalance was likely non-systemic. Adjustment was therefore deemed unnecessary and not performed.

IV effect estimates (adjusted for patient socioeconomic and admission year differences) for longer D2B delay on the STEMI outcomes are presented in Table 2. All the effect estimates appear to be directionally consistent with that of CBPS. However, widened confidence intervals are seen across all the outcome examined. Statistical significance defined at α = 0.05 was not achieved for any of these endpoints. Note that relative effects were not reported for the IV analysis. This is because the conventional 2SLS approach of IV analysis used in this study allow only the derivation of the absolute (marginal) risk difference (and the corresponding 95% CI)^14^. Relative risk reporting in IV analysis requires a different modelling approach hence was not performed.

## Discussion

Among those undergoing pPCI in Victoria, Australia during 2005 to 2015, we observed a trend indicating a consistent link between longer D2B delay and higher risk of adverse STEMI outcomes despite the added confounding consideration. This observation was based on a propensity score analysis implemented through the CBPS algorithm and an IV analysis that used patient-level “differential distance” as a pseudo-randomizer. Through the CBPS algorithm^15^, two highly comparable groups on all observed dimension were obtained. The IV analysis then further this agenda to consider the potential biases arising from the unobserved factors. These analyses were intended to consider the possibility of a confounded effect that D2B delay has on STEMI outcomes previously reported. It appears that D2B time exceeding 90 minutes is consistently associated with a higher risk of most study outcomes in the CBPS analysis. The effect estimates for all the study outcomes are still directionally consistent with that of the CBPS analysis when we further considered the IV approach, albeit a widened confidence interval that marginally covering the null.

This study also adds to the existing literature by reporting on the population average effect previously unavailable. To date, available literature lacks this important insight. Previous studies have expressed almost exclusively the effect in relative terms. As relative effect measures often yield apparently larger effects size than their absolute counterpart, healthcare managers and policy makers can be misguided if informed solely by the relative measures. Furthermore, previous D2B time-outcome studies estimated the relative effects mainly through the conventional use of logistic and Cox regression model. While these approaches offer a “conditional” effect estimate familiar and meaningful in the clinical setting, particularly for clinician-patients communication, they are of limited relevance to policy makers and population health decision makers. These group of evidence users rely more on the population average effect (also known as the marginal effect). The population average effect measure, whether in absolute or relative term, represents the change in exposure averaged across the whole population without assuming complete knowledge of the risk model, similar to those offered by a randomized trial^16^. This contrasts with the conditional effect offered by multivariate logistic and Cox regression, which is relevant only to a specific and often arbitrary substratum of individuals within the target population^11^. In this study, we reported the population average effect in both relative and absolute term to fill this current evidence gap. Based on the more precise estimated from the CBPS analysis, failure to reduce D2B time (from an average 136.9 minutes to 58.8 minutes across the whole study population) can potentially add 1.04–4.58 in-hospital deaths per 100 STEMI patients receiving pPCI. This suggest that one life might be lost by failure to optimizing D2B time in every 22 to 96 STEMI patients receiving the procedure.

### Study limitation

Despite the value and strength of this study discussed above, there are several limitations to consider. Firstly, the effect estimates captured via PS analysis and IV analysis are in fact conceptually different. In the IV analysis, the local average treatment effect is estimated. The local average treatment effect refers to the effect of the exposure on the “compliers”. Compliers in our IV analysis represent those whose D2B time experience differed according to their relative distance to hospitals with longer or shorter D2B time. In the PS analysis, the treatment effect is obtained by averaging the differences of the outcome rates across the whole study cohort^17,18^. Only when the treatment effect is homogeneous across the study population, this difference will coincide. Hence, readers should be aware that the effect estimates reported through these different analyses are likely non-identical.

Secondly, an IV analysis is limited by its underlying assumptions. The essential assumptions are that the instrument is: (1) associated with the treatment; (2) independent of all the confounding factors; and (3) independent of the outcomes given the treatment and the confounders. These assumptions can only be supported indirectly but not be verified with certainty. We demonstrated the IV strength and assessed the covariates balances in between to IV subgroups. The balances of the observed factors achieved by the IV were not as optimal as that seen in the CBPS analysis. Adjustment was made to these observed imbalances to alleviate potential residual biases. Through these adjustment, the assumptions needed to establish a reasonably valid IV analysis likely stand. Moreover, it has been suggested that, as long as these assumption are reasonably valid (need not be perfectly valid), the resulting IV estimates are still comparatively less bias^19^.

Thirdly, we realized that there are two potential sources where the instrumental variable might be misclassified: (1) the utilization of the postcode centroid as a representation of the patient’s location at event onset (vs. the actual location of the patient): Patients’ residential postcode reflect the likely location or area where he/she might be at the time of the cardiac event onset. But this is certainly less desirable compare to using the actual onset location. Unfortunately, this detailed data is unavailable to us. The mechanism of this potential misclassification is likely random; hence a systemic bias is not likely. Nevertheless, the variance of the (instrumental) variable might be increased as a result of this additional random error. (2) the use of travel distance as a representation of the proximity to the nearest hospitals (instead of other potentially more accurate indicator, e.g. the actual required travel time at event onset): Similarly, we deducted that this mechanism of misclassification will likely cause non-systemic errors to the IV classification. The key concern of a larger variance for the IV is that the IV strength might be weakened as a result. Yet, our assessment has determined that, despite the increased variance, the IV remains sufficiently powerful in predicting the exposure variable.

Lastly, the observational nature of our study precludes causal conclusions despite the intention of addressing both the observed and unobserved factors. Particularly, our results might be explained by residual confounding if the instrument is related to other unobserved factors such as treatment and facility characteristics. Evidence from our IV analysis therefore cannot exclude the presence of unmeasured confounding between the D2B time-outcome association. Further analysis is required to assess this issue. However, as the centers included in the MIG dataset are largely similar in term of these dimensions, this concern should be considered minimal.

In conclusion, a consistent linkage between longer D2B delay and higher risk of adverse STEMI outcomes is supported at the population level. These linkages persisted despite adjusting for the observed and unobserved factors potential of confounding the relation. The population average effects reported in this study indicate that failure to optimize the D2B time in pPCI can have significant negative impact on STEMI population outcomes.

## Methods

### Data sources and the study cohort

We derived the study cohort from the Melbourne Interventional Group (MIG) PCI registry^20^, a collaborative multicenter registry of Australian public referral hospitals. Details of the MIG PCI registry has been previously described^21–23^. Briefly, the baseline demographics, clinical, angiographic, and procedural characteristics of consecutive patients undergoing PCI are prospectively recorded using standardized definitions^21,22^. The study protocol was approved by the ethics committee in each participating hospital with the use of “opt-out” consent. To “opt-out” means the participant can choose to have any or all of the information about them removed from the MIG registry when indicated. In-hospital outcomes and complications were recorded at the time of discharge. Cardiac research nurses conducted 30-day follow-ups by telephone, using a standardized questionnaire. All adverse events were verified by reviewing the patients’ medical records at the relevant hospitals. The Centre for Cardiovascular Research & Education in Therapeutics, a research body within the Department of Epidemiology and Preventive Medicine, Monash University coordinated the registry. An independent audit is routinely conducted at all enrolling sites by an investigator not affiliated with that institution. Data accuracy was 97%^24^.

We included adult patients (≥18 years) with STEMI admitted to one of the six participating hospitals in Victoria, Australia during 2005–2015. We excluded patients with D2B time of greater than 6 hours. This study was approved by the Monash University Human Research Ethics Committee (MUHREC Project number: CF15/4503-2015001952) who determined that this study satisfied section 5.1.22 of the Australia National Statement on Ethical Conduct in Human Research. The MIG registry steering committee permitted the conduct of this study after the MUHREC review exemption. Reporting of this study is in accordance to the Strengthening the Reporting of Observational studies in Epidemiology guideline. (See Appendix A-1).

### Study context

There are 13 public hospitals and 18 private hospitals across all areas of Victoria, Australia. STEMI patients in Victoria have been predominantly treated in the public sector (~90% of all STEMI cases), with close to 90% of the pPCI being performed in the public hospital system^25^. Emergency responses, pre-hospital cares and inter-hospital transfers of acute patients across the public and private system, including when a cardiac condition is suspected, are delivered by Ambulance Victoria. A patient located in the metropolitan with a suspected STEMI will typically be taken to the nearest emergency department (of a public hospital) at the time of onset. The pre- and in-hospital processes & management of STEMI in Victoria for the public hospitals are standardized across regions of Victoria. Patients who are privately insured and located within the inner city may opt for a private facility for their STEMI care and be transferred accordingly. However, scenario as such are considered the minority. A geo-graphical illustration of the cardiac centers distribution in Victoria is provided in Appendix A-2. More general information on the Australian health system can be referred to here^26^.

The MIG registry dataset used in this study was derived from six public hospitals that provided specialist cardiac services for adult patients^20^: The Alfred, Austin Hospital, University Hospital Geelong, The Royal Melbourne Hospital, Box Hill Hospital and Ballarat Health Services. These six public centers represented close to 40% of the overall primary PCI caseload within the Victoria state. Most of the PCI procedures performed in the private hospitals were elective cases. Slight differences have been observed in between the STEMI patients treated by the public cardiac centers and those treated in the private hospitals: STEMI patients in the private sector were observed to be slightly older (66.3 ± 12.1 private sector vs 62.7 ± 12.4 public sector), but otherwise had similar gender and risk profiles as those treated in the public centers^25^.

### Exposures and outcome variables

The exposure variable under study was the patient-level D2B time. D2B time refers to the time from a patient’s arrival to a pPCI capable hospital to the first device use during pPCI. Examples of the first device used include, but are not limited to balloon, thrombectomy device, atherectomy device or stent. When the lesion failed to be crossed by the guidewire or device, the device time was taken as the time that the guide catheter was initially introduced. We examined the effect of D2B time has on STEMI outcomes by considering D2B time as a dichotomized variable: ≤90 minutes vs. >90 minutes.

Primary outcomes assessed included in-hospital and 30-day mortality. In-hospital shock, arrhythmia and in-hospital and 30-day composite endpoints (major adverse cardiac event, MACE) were considered as secondary outcomes. Shock is referred if the patient suffered a new episode or acute recurrence of cardiogenic shock following the PCI procedure. (See Appendix A-2). Arrhythmia refers to a new episode or acute recurrence of an atrial or ventricular arrhythmia requiring treatment or a new episode of high-level atrioventricular block. In-hospital MACE is defined as the presence of in-hospital shock, arrhythmia or death; 30-day MACE is defined as the presence of any of the following: in-hospital MACE, readmission and 30-day death.

Mortality data (30-day) were obtained by linkage to the Australian National Death Index^23^. The Australian National Death Index is a database that contains records of all deaths occurring in Australia since 1980. Successful matching of patients through this linkage process was achieved in 99.42% of patients in the MIG registry.

### Other variables

We assessed the baseline characteristic of the whole study cohort and the differences in patient characteristics by D2B delay. Demographic variables included age, sex, and race at the time of the procedure. The Socio-Economic Indexes for Areas^27^ developed by the Australian Bureau of Statistics and the Cardiac Accessibility and Remoteness Index for Australia^28^ were used to represent a patient’s socioeconomic status and health service accessibility based on their place of residence. We also assessed patients’ comorbidities, pre-hospital delay, day-time factors of STEMI onset and angiographic variables.

### Analytic approaches

#### Covariate balancing propensity score analysis

Propensity score is the estimated probability of a study subject experiencing an exposure^29^. Weighting the subjects based on their propensity score create a pseudo-cohort with balance covariates in between the comparison groups^29^. In this study, we modelled the PS using the CBPS approach^30^. This approach allows the simultaneous maximization the resulting covariate balance as well as the prediction of treatment assignment, thereby avoiding an iteration between model fitting and balance checking. Covariate balance after CBPS weighting was evaluated by comparing the weighted covariates in between the exposure groups. The list of predictors included in the CBPS model is provided in Appendix A-5.

The adjusted risk of the study outcomes for each exposure group were calculated by weighting the outcomes using the CBPS weights. Comparison of the weighted risks in between the exposure groups were made; the population average relative risk and absolute risk differences were reported. The corresponding bootstrapped (200 iterations) 95% confidence intervals were provided. The CBPS weights were re-estimated in each iteration of bootstrapping.

#### Instrumental variable analysis

An “instrument” is used in an IV analysis to achieve a natural randomization that minimizes the biases from both the measured and unmeasured confounders^14,31,32^. There are several common sources of IV for comparative studies in medicine. The potential sources of IV include: (variation of) physician treatment preference, exposure time, and natural genetic variants^19^. Distance has been another commonly considered variable that poses features of a suitable instrument, particularly in condition of emergent nature (like STEMI). This is because proximity of a patient to an emergency care provider (i.e. distance) generally enhances the likelihood of him/her being treated by the specific care provider. For less acute conditions, patients and providers will have more time to plan and decide where to be better treated. Hence proximity may have lesser influence on treatment selection/exposure. McClellan *et al*.’s study on cardiac catheterization effects on STEMI outcomes represents a classic example of a distance-based IV analysis under an emergent condition^33^. The IV used in McClellan’s study was the “differential distance” (DD) the patient lives from the nearest hospital that performs cardiac catheterization to the nearest hospital that does not perform cardiac catheterization. Because distance to a specialty care provider is often associated with socioeconomic characteristics, it is often necessary to control for the socioeconomic characteristics in order for distance to potentially be independent of the unmeasured confounders. Additionally, the possibility that distance might have a direct effect on outcomes (because the time taken to receive treatment may have the potential to affect outcomes) also will required explicit consideration in order to ensure the high validity of the IV.

Our analysis took reference of McClellan *et al*.’s study and several other similar studies^34,35^. We used the DD as an instrument to achieve a pseudo-randomization objective as per above description. DD of a STEMI patient in this study is referred to as the additional distance one has to travel beyond his or her nearest hospital in order to reach another hospital with a shorter (median) D2B time (see Appendix A-3 for the conceptual illustration). A positive DD means that the patient is relatively farther away from a hospital with shorter D2B time; a negative DD means otherwise. Patients with STEMI who arrive at a hospital with shorter median D2B time are more likely to experience a timely reperfusion. Moreover, differential distance is unlikely to be related to patients’ outcome independently except through the better chance of experiencing a shorter D2B time given that the socioeconomic variables are adjusted.

We used the “geocode” function from the ggmap package^25^ of R to obtain the longitude and latitude of each patient’s location based on the postcode of their residential address. The geocode function operated by interfacing with the Google Geocoding API, which call for the longitude and latitude value of each input residential postcode area (the centroid of the postcode area) from the google map. A similar process was applied to obtaining the longitude and latitude data for each of the registry hospital. To derive the driving distances from each patient’s geocoded location to their two nearest hospitals, we used the “gmapsdistance” function. This function uses the Google Maps Distance Matrix API to compute the (driving) distance between two points on the globe.

After obtaining the distance data from the above process, differential distance was computed as per the description provided above. From there, we excluded those who resided in zip codes where the absolute DD was less than 5 kilometers to improve the instrumental strength. We then dichotomized the DD into two groups. DD >5 km refers to those whose residential location were comparatively farther away from a hospital with shorter median D2B time; DD <−5 km refers to the opposite.

Estimation of the effect of D2B time reduction using the differential distance as a randomizer were implemented through a 2-stage least square (2SLS) model^14,19^. The strength of the instrument is assessed during the first stage by observing the F-statistic of the least square model. An F-statistic of greater than 10 is considered an adequately strong instrument^19,36^. The assumption that the instrument is unrelated to the study outcomes except through the exposure is assessed by comparing the observed differences in between the 2 differential distance groups^11^. A balance of observed covariates in between the two differential distance groups suggest that this assumption is reasonably valid. Robust standard errors were used. The absolute risk differences on the complier sub-population is reported. Reporting standards previously published were used^19,36^. (See Appendix A-4).

### Missing data and imputation

To allow for the uncertainty arising from missing data, we used the multiple imputation technique^37^. The AMELIA II imputation package^38^ was used which implements a bootstrapping-based algorithm using the expectation–maximization and Bayesian hierarchical classification model. Five copies of the dataset with the missing values were replaced by imputed values. The CBPS analysis were executed to each of the imputed datasets. An overall estimate (with standard error) were obtained by applying the Rubin’s rules^39^. Imputed datasets were not used for the IV analysis as variables needed for the IV analysis contains no missing data.

All analyses were performed using R version 3.3.1 and evaluated at a 2-sided significance level of p < 0.05. sized differences (SMD) of covariates of ≤0.1 (i.e. one-tenth of a standard deviation apart) between the comparison groups are considered negligible. The 2SLS models were implemented using the ivpack package^40^ and the CBPS package^15^ for CBPS analysis.

## Supplementary information

Supplementary Materials (A1-A7)

## Data Availability

The data that support the findings of this study are available from the Melbourne Interventional Group (MIG) PCI registry. But restrictions apply to the availability of these data, as this health information is considered sensitive information under the Privacy Act, hence they are not publicly available.
