# Supplementary material for: Re-examining the effect of door-to-balloon delay on STEMI outcomes in the context of unmeasured confounders: a retrospective cohort study
Source: Sci Rep. 2019 Dec 27;9:19978. doi: 10.1038/s41598-019-56353-7 (PMC6934575; doi:10.1038/s41598-019-56353-7)
Supplement: Supplementary file 1 — Supplementary Materials (A1-A7) [file 41598_2019_56353_MOESM1_ESM.docx]

**Supplementary Material** Version 22 November, 2019

Re-examining the effect of door-to-balloon delay on STEMI outcomes in the context of unmeasured confounders: a retrospective cohort study

*Chee Yoong Foo1,2,3 researcher & PhD candidate, Nick Andrianopoulos4 adjunct senior research fellow, Angela Brennan5 senior research fellow, Andrew Ajani6 associate professor, Christopher M Reid4,7 professor, Stephen J Duffy5,8 professor, David J Clark9 associate professor, Daniel D Reidpath3,11,12 professor, Nathorn Chaiyakunapruk2,13,14,15 professor*

*^1^National Clinical Research Centre, Malaysia. ^2^School of Pharmacy,* *Monash University Malaysia, Bandar Sunway, Selangor, Malaysia. ^3^Jeffrey Cheah School of Medicine and Health Sciences, Monash University Malaysia, Bandar Sunway, Selangor, Malaysia. ^4^School of Epidemiology and Preventive Medicine, Monash University, Melbourne, VIC, Australia. ^5^Centre of Cardiovascular Research & Education in Therapeutics, Department of Epidemiology and Preventive Medicine, School of Public Health and Preventive Medicine, Monash University, Melbourne, VIC, Australia.* ^6^*Department of Cardiology, Royal Melbourne Hospital, Melbourne, Australia; Department of Medicine, University of Melbourne, Melbourne, Australia; Centre of Cardiovascular Research and Education in Therapeutics (CCRET), Monash University, Melbourne, Australia.* ^7^*School of Public Health, Curtin University, Perth, WA, Australia. ^8^Department of Cardiology, Austin Hospital, Melbourne, Australia. ^9^Department of Cardiology, Alfred Hospital, Melbourne, Australia. ^10^Faculty of Pharmaceutical Sciences, Naresuan University, Phitsanulok, Thailand. ^11^School of Population Health, Curtin University, Perth, Australia. ^12^Molecular, Genetic & Population Health Sciences, University of Edinburgh, Edinburgh, UK. ^13^Center of Pharmaceutical Outcomes Research (CPOR), Department of Pharmacy Practice, Faculty of Pharmaceutical Sciences, Naresuan University, Phitsznulok, Thailand. ^14^School of Pharmacy, University of Wisconsin, Madison, USA. ^15^Health and well-being cluster, Global Asia Platform, Monash University Malaysia*

Contents

[A-1 STROBE Statement 2](#_Toc523501652)

[A-2 Clinical criteria for cardiogenic shock 5](#_Toc523501653)

[A-3 Differential distance (instrumental variable) - conceptual illustration & deriving algorithm 6](#_Toc523501654)

[A-4 Published standards of IV analysis reporting 7](#_Toc523501655)

[A-5 Details of study cohort characteristics and (im)balance 8](#_Toc523501656)

[A-6 Details of covariate balance for crude, CBPS weighted and IV analysis 11](#_Toc523501657)

[A-7 Visualization of covariate balance for crude, CBPS weighted and IV analysis 15](#_Toc523501658)

# A-1 STROBE Statement

Checklist of items that should be included in reports of ***cohort studies***

|  | Item No | Recommendation | Check | Location |
| --- | --- | --- | --- | --- |
| **Title and abstract** | 1 | (*a*) Indicate the study’s design with a commonly used term in the title or the abstract | ✓ |  |
|  |  | (*b*) Provide in the abstract an informative and balanced summary of what was done and what was found | ✓ |  |
| Introduction | | |  |  |
| Background/rationale | 2 | Explain the scientific background and rationale for the investigation being reported | ✓ |  |
| Objectives | 3 | State specific objectives, including any prespecified hypotheses | ✓ |  |
| Methods | | |  |  |
| Study design | 4 | Present key elements of study design early in the paper | ✓ |  |
| Setting | 5 | Describe the setting, locations, and relevant dates, including periods of recruitment, exposure, follow-up, and data collection | ✓ |  |
| Participants | 6 | (*a*) Give the eligibility criteria, and the sources and methods of selection of participants. Describe methods of follow-up | ✓ |  |
|  |  | (*b*) For matched studies, give matching criteria and number of exposed and unexposed | Not applicable |  |
| Variables | 7 | Clearly define all outcomes, exposures, predictors, potential confounders, and effect modifiers. Give diagnostic criteria, if applicable | ✓ |  |
| Data sources/ measurement | 8* | For each variable of interest, give sources of data and details of methods of assessment (measurement). Describe comparability of assessment methods if there is more than one group | ✓ |  |
| Bias | 9 | Describe any efforts to address potential sources of bias | ✓ |  |
| Study size | 10 | Explain how the study size was arrived at | ✓ |  |
| Quantitative variables | 11 | Explain how quantitative variables were handled in the analyses. If applicable, describe which groupings were chosen and why | ✓ |  |
| Statistical methods | 12 | (*a*) Describe all statistical methods, including those used to control for confounding | ✓ |  |
|  |  | (*b*) Describe any methods used to examine subgroups and interactions | Not applicable |  |
|  |  | (*c*) Explain how missing data were addressed | ✓ |  |
|  |  | (*d*) If applicable, explain how loss to follow-up was addressed | Not applicable |  |
|  |  | (*e*) Describe any sensitivity analyses | Not applicable |  |
| Results | | |  |  |
| Participants | 13* | (a) Report numbers of individuals at each stage of study—eg numbers potentially eligible, examined for eligibility, confirmed eligible, included in the study, completing follow-up, and analysed | ✓ |  |
|  |  | (b) Give reasons for non-participation at each stage | ✓ |  |
|  |  | (c) Consider use of a flow diagram | ✓ |  |
| Descriptive data | 14* | (a) Give characteristics of study participants (eg demographic, clinical, social) and information on exposures and potential confounders | ✓ |  |
|  |  | (b) Indicate number of participants with missing data for each variable of interest | ✓ |  |
|  |  | (c) Summarise follow-up time (eg, average and total amount) | Not applicable |  |
| Outcome data | 15* | Report numbers of outcome events or summary measures over time | Not applicable |  |
| Main results | 16 | (*a*) Give unadjusted estimates and, if applicable, confounder-adjusted estimates and their precision (eg, 95% confidence interval). Make clear which confounders were adjusted for and why they were included | ✓ |  |
|  |  | (*b*) Report category boundaries when continuous variables were categorized | ✓ |  |
|  |  | (*c*) If relevant, consider translating estimates of relative risk into absolute risk for a meaningful time period | ✓ |  |
| Other analyses | 17 | Report other analyses done—eg analyses of subgroups and interactions, and sensitivity analyses | No other analysis |  |
| Discussion | | |  |  |
| Key results | 18 | Summarise key results with reference to study objectives | ✓ |  |
| Limitations | 19 | Discuss limitations of the study, taking into account sources of potential bias or imprecision. Discuss both direction and magnitude of any potential bias | ✓ |  |
| Interpretation | 20 | Give a cautious overall interpretation of results considering objectives, limitations, multiplicity of analyses, results from similar studies, and other relevant evidence | ✓ |  |
| Generalisability | 21 | Discuss the generalisability (external validity) of the study results | ✓ |  |
| Other information | | |  |  |
| Funding | 22 | Give the source of funding and the role of the funders for the present study and, if applicable, for the original study on which the present article is based | ✓ |  |

# A-2 Clinical criteria for cardiogenic shock

Clinical criteria for cardiogenic shock are:

- Hypotension (a systolic blood pressure of less than 90 mmHg for at least 30 minutes or the need for supportive measures to maintain a systolic blood pressure of greater than or equal to 90mmHg)
- End-organ hypoperfusion (cool extremities or a urine output of less than 30 ml/hour, and a heart rate of greater than or equal to 60 beats per minute).
- The hemodynamics criteria are a cardiac index of no more than 2.2 l/min per square meter of body-surface area and a pulmonary-capillary wedge pressure of at least 15 mmHg.

# A-3 Differential distance (instrumental variable) - conceptual illustration & deriving algorithm

**A-3-1. Geographical distribution of cardiac centres in central Victoria - Public (PCI-capable & non-PCI capable) and Private centres.** Map generated using the ggmap package* (version 3.0.0) of R version 3.5.1


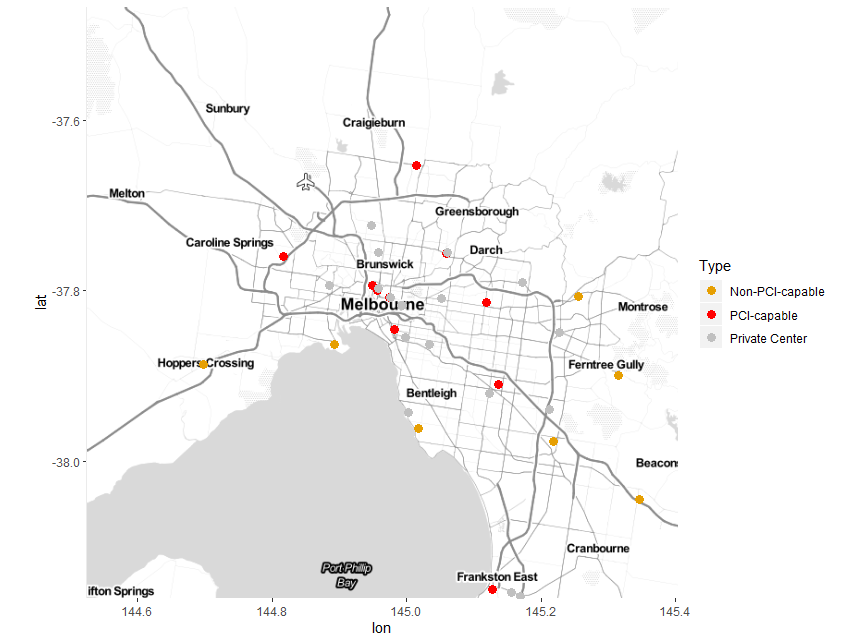
*Kahle D, Wickham H. ggmap: Spatial Visualization with ggplot2. The R Journal 2013;5(1):144-61. doi: 10.32614/RJ-2013-014

**A-3-2.
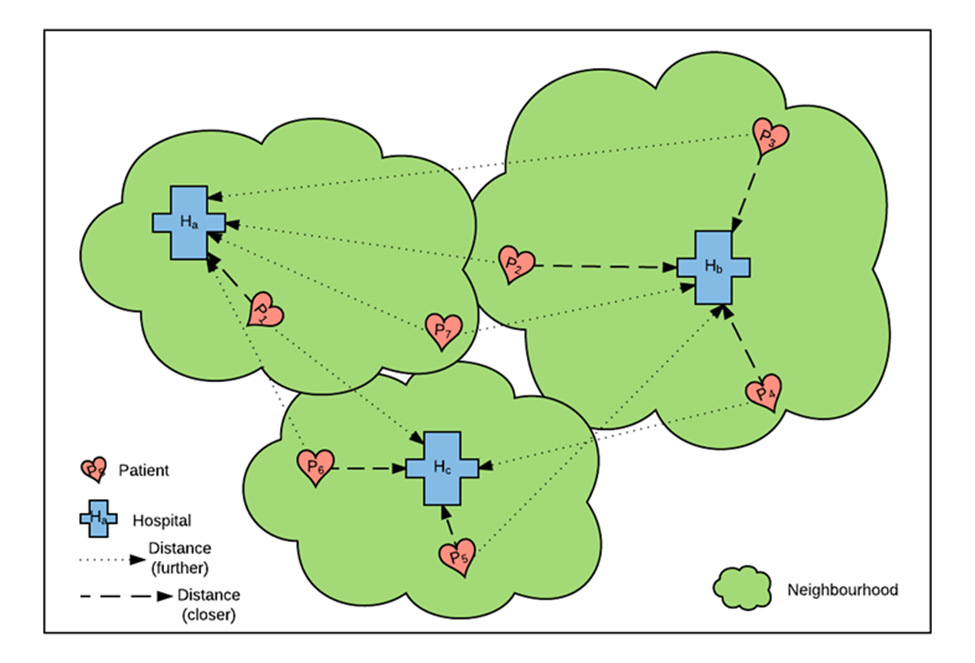
Conceptual illustration**

| **A-3-3. Derivation algorithm of the instrumental variable - differential distance** | |
| --- | --- |
| 1. Obtain the coordinate of a patient location (using the centroid of the patient’s residential postcode) | |
| 1. Identify the coordinate of all hospitals located in the study area | |
| 1. For each patient's location, find the two nearest PCI-capable hospital by travel distance | |
| 1. Calculate the travel distance to these two hospitals | |
| 1. Obtain the annual median D2B time of the two nearest hospitals for each patient | |
| 1. Subtract A from B.   A = the distance between a patient location to the shorter median D2B time hospital; B = the distance between a patient location to the longer median D2B time hospital.  Hence, the instrument, i.e. the differential distance (DD) = A-B. | |
| - If DD = 0, no difference in term of the tendency toward experiencing a timelier reperfusion; if DD < 0 = more likely to experience timely reperfusion; if DD > 0 = more likely to experience a delayed reperfusion)   See below (A-3-4) illustration to further understand the IV derivation within the study setting. | |


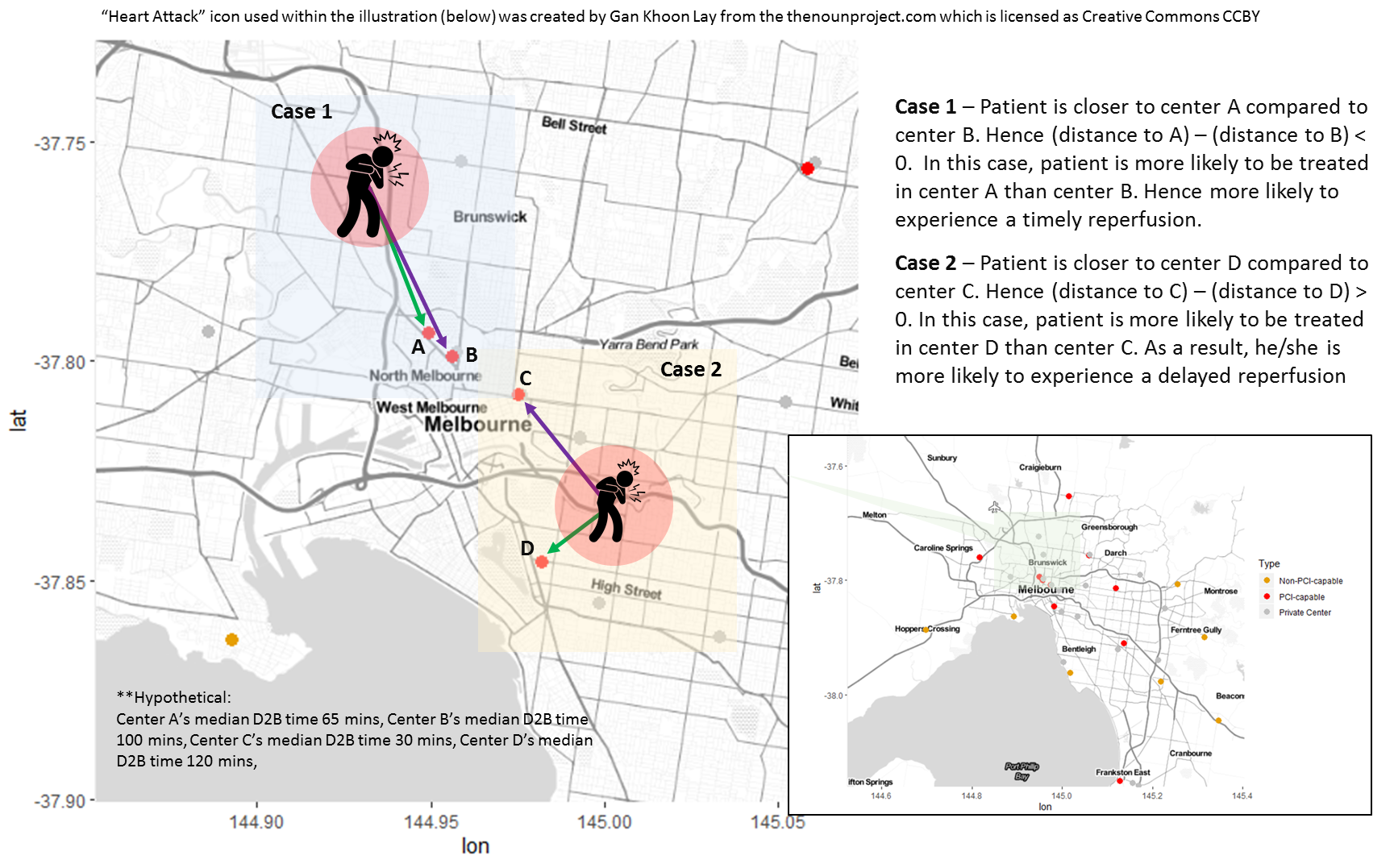
**A-3-4. Illustrative cases with graphical aids to depict the IV derivation.** Map generated using the ggmap package* (version 3.0.0) of R version 3.5.1

*Kahle D, Wickham H. ggmap: Spatial Visualization with ggplot2. The R Journal 2013;5(1):144-61. doi: 10.32614/RJ-2013-014

# A-4 Published standards of IV analysis reporting

| **No.** | **Davies 2013^1^** | **Checked** |
| --- | --- | --- |
| 1 | State the population target parameter | √ |
| 2 | State the assumptions | √ |
| 3 | Report the association of the instrument and exposure using a partial F-statistic | √ |
| 4 | Report and test the association of observed potential confounding factors with both the exposure and the instrument | √ |
| 5 | With multiple instruments, report the test for over-identifying restrictions | NA |
| 6 | For binary outcomes, exposures and instruments, report a tabulation of the frequencies of each combination of instrument, exposure and outcome, so readers can reconstruct basic results | √ |
| 7 | When using generalized linear models with binary outcomes, always use robust or bootstrapped standard errors and take clustering of study participants into account where necessary | √ |

**^1^**Davies NM, Smith GD, Windmeijer F, Martin RM. Issues in the reporting and conduct of instrumental variable studies: a systematic review. Epidemiology. 2013;24(3):363-9.

| **No.** | **Baiocchi 2014^2^** | **Checked** |
| --- | --- | --- |
| 1 | Describe theoretical basis for choice of IV | √ |
| 2 | Report strength of IV and results from first-stage model | √ |
| 3 | Report distribution of measured confounders across levels of the IV and treatment | √ |
| 4 | Explore concomitant treatments | √ |
| 5 | Discuss the interpretation of the treatment effect estimated by the IV | √ |
| 6 | Report a sensitivity analysis | NA |

^2^Baiocchi M, Cheng J, Small DS. Instrumental variable methods for causal inference. Stat Med. 2014;33(13):2297-340.

# A-5 Details of study cohort characteristics and (im)balance

|  |  |  | **Overall** | **≤ 90 mins** | **> 90 mins** | **Std. Diff** |
| --- | --- | --- | --- | --- | --- | --- |
| No. of patients | | | *N=4343* | *n=2692* | *n=1651* |  |
| Age, mean (sd) | | | 63.29 (12.81) | 62.79 (12.47) | 64.09 (13.31) | 0.101 |
| Male, n (%) | | | 3398 (78.2) | 2152 (79.9) | 1246 (75.5) | 0.108 |
| Race, n (%) | | |  |  |  | 0.111 |
|  | Caucasian | | 4000 (92.1) | 2483 (92.2) | 1517 (91.9) |  |
|  | Asian | | 131 ( 3.0) | 78 ( 2.9) | 53 ( 3.2) |  |
|  | Other | | 179 (4.1) | 129 (4.8) | 79 (4.8) |  |
|  | Missing | | 4 ( 0.1) | 2 ( 0.1) | 2 ( 0.1) |  |
| BMI, mean (sd) | | | 27.70 (4.91) | 27.72 (4.75) | 27.68 (5.17) | 0.008 |
| Smoking history, n (%) | | |  |  |  | 0.09 |
|  | Unknown | | 61 ( 1.4) | 31 ( 1.2) | 30 ( 1.8) |  |
|  | Current | | 1545 (35.6) | 988 (36.7) | 557 (33.7) |  |
|  | Prior | | 1205 (27.7) | 751 (27.9) | 454 (27.5) |  |
|  | Never | | 1502 (34.6) | 906 (33.7) | 596 (36.1) |  |
|  | Missing | | 30 ( 0.7) | 16 ( 0.6) | 14 ( 0.8) |  |
| Congestive heart failure (within 2 weeks), n (%) | | | 259 (6.0) | 130 (4.8) | 129 (7.8) | 0.133 |
|  | Missing | | 2 (0.0) | 0 (0.0) | 2 (0.1) |  |
| Family history of CAD, n (%) | | | 2747 (63.3) | 1695 (63.0) | 1052 (63.7) | 0.096 |
|  | Unknown | | 135 ( 3.1) | 86 ( 3.2) | 49 ( 3.0) |  |
|  | Missing | | 47 ( 1.1) | 19 ( 0.7) | 28 ( 1.7) |  |
| Pre-procedural TIMI flow | | |  |  |  | 0.193 |
|  | 1 |  | 3053 (70.3) | 1976 (73.4) | 1077 (65.2) |  |
|  | 2 |  | 244 ( 5.6) | 149 ( 5.5) | 95 ( 5.8) |  |
|  | 3 |  | 421 ( 9.7) | 231 ( 8.6) | 190 (11.5) |  |
|  | 4 |  | 620 (14.3) | 335 (12.4) | 285 (17.3) |  |
|  | Missing | | 5 ( 0.1) | 1 ( 0.0) | 4 ( 0.2) |  |
| NYHA class | | |  |  |  | 0.101 |
|  | Class I | | 94 ( 2.2) | 53 ( 2.0) | 41 ( 2.5) |  |
|  | Class II | | 2309 (53.2) | 1465 (54.4) | 844 (51.1) |  |
|  | Class III | | 341 ( 7.9) | 206 ( 7.7) | 135 ( 8.2) |  |
|  | Class IV | | 141 ( 3.2) | 88 ( 3.3) | 53 ( 3.2) |  |
|  | Not recorded | | 812 (18.7) | 511 (19.0) | 301 (18.2) |  |
|  | Missing | | 646 (14.9) | 369 (13.7) | 277 (16.8) |  |
| Killip class | | |  |  |  | 0.146 |
|  | 1 |  | 3160 (72.8) | 2014 (74.8) | 1146 (69.4) |  |
|  | 2 |  | 463 (10.7) | 272 (10.1) | 191 (11.6) |  |
|  | 3 |  | 100 ( 2.3) | 48 ( 1.8) | 52 ( 3.1) |  |
|  | 4 |  | 352 ( 8.1) | 197 ( 7.3) | 155 ( 9.4) |  |
|  | Not recorded | | 60 ( 1.4) | 31 ( 1.2) | 29 ( 1.8) |  |
|  | Missing | | 208 ( 4.8) | 130 ( 4.8) | 78 ( 4.7) |  |
| Out of hospital cardiac arrest, n (%) | | | 461 (10.6) | 257 (9.5) | 204 (12.4) | 0.09 |
| Systolic BP, mean (sd) | | | 127.98 (27.67) | 128.84 (27.77) | 126.12 (27.39) | 0.099 |
| Cardiogenic shock (pre-procedure), n (%) | | | 465 (10.7) | 248 (9.2) | 217 (13.1) | 0.125 |
|  |  |  |  |  |  |  |
| **Time-day factors** | | |  |  |  |  |
|  | Weekend admission, n (%) | | 1222 (28.1) | 664 (24.7) | 558 (33.8) | 0.202 |
|  | Off-hour presentation, n (%) | | 2075 (47.8) | 1120 (41.6) | 955 (57.8) | 0.333 |
|  | Onset-to-door-time, mean (sd) | | 154.11 (176.33) | 147.7 (158.7) | 165.7 (204.18) | 0.098 |
|  |  | Missing | 356 ( 8.2) | 117 ( 4.3) | 239 ( 14.4) |  |
|  | Door-to-balloon time, mean (sd) | | 88.5 (51.4) | 58.8 (19.2) | 136.9 (50.9) | 2.031 |
|  |  |  |  |  |  |  |
| **Socio-economics & healthcare accessibility** | | |  |  |  |  |
|  | Cardiac ARIA index | |  |  |  | 0.105 |
|  |  | Zone 1A | 3985 (91.8) | 2444 (90.8) | 1541 (93.3) |  |
|  |  | Zone 2A | 180 (4.1) | 119 (4.4) | 61 (3.7) |  |
|  |  | Zone 3A & 4A | 96 (2.2) | 69 (2.6) | 27 (1.6) |  |
|  |  | Zone 5A and above | 77 (1.8) | 57 (2.1) | 20 (1.2) |  |
|  |  | Missing | 5 (0.1) | 3 (0.1) | 2 (0.1) |  |
|  | IRSAD (%) - Socio-economic | |  |  |  | 0.119 |
|  |  | Lowest - 1st quartile | 1089 (25.1) | 667 (24.8) | 422 (25.6) |  |
|  |  | 1st quartile - median | 1123 (25.9) | 746 (27.7) | 377 (22.8) |  |
|  |  | Median - 3rd quartile | 1066 (24.5) | 652 (24.2) | 414 (25.1) |  |
|  |  | 3rd quartile - highest | 1065 (24.5) | 627 (23.3) | 438 (26.5) |  |
|  | IRSD (%) - Socio-economic | |  |  |  | 0.091 |
|  |  | Lowest - 1st quartile | 1132 (26.1) | 678 (25.2) | 454 (27.5) |  |
|  |  | 1st quartile - median | 1087 (25.0) | 705 (26.2) | 382 (23.1) |  |
|  |  | Median - 3rd quartile | 1038 (23.9) | 657 (24.4) | 381 (23.1) |  |
|  |  | 3rd quartile - highest | 1086 (25.0) | 652 (24.2) | 434 (26.3) |  |
|  | IER (%) - Economic resources | |  |  |  | 0.123 |
|  |  | Lowest - 1st quartile | 1090 (25.1) | 626 (23.3) | 464 (28.1) |  |
|  |  | 1st quartile - median | 1091 (25.1) | 677 (25.1) | 414 (25.1) |  |
|  |  | Median - 3rd quartile | 1156 (26.6) | 730 (27.1) | 426 (25.8) |  |
|  |  | 3rd quartile - highest | 1006 (23.2) | 659 (24.5) | 347 (21.0) |  |
|  | IEO (%) - Education & occupation | |  |  |  | 0.123 |
|  |  | Lowest - 1st quartile | 1144 (26.3) | 720 (26.7) | 464 (28.1) |  |
|  |  | 1st quartile - median | 1058 (24.4) | 716 (26.6) | 414 (25.1) |  |
|  |  | Median - 3rd quartile | 1074 (24.7) | 668 (24.8) | 426 (25.8) |  |
|  |  | 3rd quartile - highest | 1067 (24.6) | 588 (21.8) | 347 (21.0) |  |
|  |  |  |  |  |  |  |
| **Co-morbidities** | | |  |  |  |  |
|  | Renal transplant, n (%) | | 10 (0.2) | 8 (0.3) | 2 (0.1) | 0.04 |
|  |  | Missing | 9 (0.2) | 5 (0.2) | 4 (0.2) |  |
|  | Obstructive sleep apnea, n (%) | | 88 (2.0) | 52 (1.9) | 36 (2.2) | 0.026 |
|  |  | Missing | 13 (0.3) | 7 (0.3) | 6 (0.4) |  |
|  | Rheumatoid arthritis, n (%) | | 75 (1.7) | 49 (1.8) | 26 (1.6) | 0.03 |
|  |  | Missing | 11 (0.3) | 8 (0.3) | 3 (0.2) |  |
|  | Cerebrovascular disease, n (%) | | 209 (4.8) | 114 (4.2) | 95 (5.8) | 0.089 |
|  |  | Missing | 9 (0.2) | 8 (0.3) | 1 (0.1) |  |
|  | Dyslipidemia, n (%) | | 2124 (48.9) | 1285 (47.7) | 839 (50.8) | 0.063 |
|  |  | Missing | 13 (0.3) | 9 (0.3) | 4 (0.2) |  |
|  | Chronic lung disease, n (%) | | 361 (8.3) | 200 (7.4) | 161 (9.8) | 0.083 |
|  |  | Missing | 15 (0.3) | 9 (0.3) | 6 (0.4) |  |
|  | Asthma, n (%) | | 181 (4.2) | 107 (4.0) | 74 (4.5) | 0.025 |
|  | COPD, n (%) | | 184 (4.2) | 96 (3.6) | 88 (5.3) | 0.086 |
|  | Peripheral vascular disease, n (%) | | 2124 (48.9) | 1285 (47.7) | 839 (50.8) | 0.063 |
|  |  | Missing | 13 (0.3) | 9 (0.3) | 4 (0.2) |  |
|  | Hypertension, n (%) | | 2300 (53.0) | 1389 (51.6) | 911 (55.2) | 0.072 |
|  |  | Missing | 8 (0.2) | 5 (0.2) | 3 (0.2) |  |
|  | Diabetes, n (%) | | 748 (17.2) | 443 (16.5) | 305 (18.5) | 0.055 |
|  |  | Missing | 7 (0.2) | 5 (0.2) | 2 (0.1) |  |
|  | Diabetes on diet modification, n (%) | | 167 (3.8) | 101 (3.8) | 66 (4.0) | 0.013 |
|  | Diabetes on oral hypoglycemic, n (%) | | 454 (10.5) | 271 (10.1) | 183 (11.1) | 0.033 |
|  | Diabetes on insulin, n (%) | | 160 (3.7) | 91 (3.4) | 69 (4.2) | 0.042 |
|  | Previous MI, n (%) | | 553 (12.7) | 305 (11.3) | 248 (15.0) | 0.11 |
|  |  | Missing | 9 (0.2) | 5 (0.2) | 4 (0.2) |  |
|  | Congestive heart failure, n (%) | | 73 (1.7) | 30 (1.1) | 43 (2.6) | 0.111 |
|  |  | Missing | 9 (0.2) | 6 (0.2) | 3 (0.2) |  |
|  | Previous PCI, n (%) | | 470 (10.8) | 0.10 (0.30) | 0.12 (0.33) | 0.088 |
|  |  | Missing | 4 (0.1) | 4 (0.1) | 0 (0.0) |  |
|  | Previous CABG, n (%) | | 97 (2.2) | 47 (1.7) | 50 (3.0) | 0.092 |
|  |  | Missing | 2 (0.0) | 2 (0.1) | 0 (0.0) |  |
|  | Previous valvular surgery, n (%) | | 16 (0.4) | 6 (0.2) | 10 (0.6) | 0.081 |
|  |  | Missing | 4 (0.1) | 4 (0.1) | 0 (0.0) |  |
|  |  |  |  |  |  |  |
| **Angiographic characteristics** | | |  |  |  |  |
|  | Lesion type, n (%) | |  |  |  | 0.071 |
|  |  | Type A | 174 (4.0) | 111 (4.1) | 63 (3.8) |  |
|  |  | Type B1 | 904 (20.8) | 556 (20.7) | 348 (21.1) |  |
|  |  | Type B2 | 2085 (48.0) | 1319 (49.0) | 766 (46.4) |  |
|  |  | Type C | 1179 (27.1) | 706 (26.2) | 473 (28.6) |  |
|  |  | Missing | 1 (0.0) | 0 (0.0) | 1 (0.1) |  |
|  | Left main lesion | | 37 (0.9) | 14 (0.5) | 23 (1.4) | 0.09 |
|  | Right coronary lesion | | 1794 (41.3) | 1233 (45.8) | 561 (34.0) | 0.243 |
|  | Left anterior descending lesion | | 1781 (39.6) | 1018 (37.8) | 700 (42.4) | 0.094 |
|  | Left anterior descending proximal lesion | | 958 (22.1) | 563 (20.9) | 395 (23.9) | 0.072 |
|  | Circumflex lesion | | 466 (10.7) | 252 (9.4) | 214 (13.0) | 0.115 |
|  | Diagonal branch lesion | | 229 (5.3) | 136 (5.1) | 93 (5.6) | 0.026 |
|  | Obtuse marginal branch lesion | | 223 (5.1) | 106 (3.9) | 117 (7.1) | 0.138 |
|  | Saphenous vein graft lesion | | 26 (0.6) | 14 (0.5) | 12 (0.7) | 0.026 |
|  | Bypass graft lesion | | 35 (0.8) | 18 (0.7) | 17 (1.0) | 0.039 |
|  | Transient or persistent no reflow | | 64 (1.5) | 38 (1.4) | 26 (1.6) | 0.013 |
|  | Total stent length >=20mm | | 1820 (41.9) | 1171 (43.5) | 649 (39.3) | 0.085 |
|  | Reference vessel diameter <=2.5mm | | 837 (19.3) | 430 (16.0) | 407 (24.7) | 0.217 |
|  | Proximal lesion | | 1996 (46.0) | 1247 (46.3) | 749 (45.4) | 0.019 |
|  | Bifurcated lesion | | 486 (11.2) | 281 (10.4) | 205 (12.4) | 0.062 |
|  | Chronic total occlusion | | 59 (1.4) | 34 (1.3) | 25 (1.5) | 0.021 |
|  | Number of stents in procedure | |  |  |  | 0.141 |
|  |  | 1 | 280 ( 6.4) | 144 ( 5.3) | 136 ( 8.2) |  |
|  |  | 2 | 3161 (72.8) | 2001 (74.3) | 1160 (70.3) |  |
|  |  | 3 | 737 (17.0) | 435 (16.2) | 302 (18.3) |  |
|  |  | 4 or more | 165 ( 3.8) | 112 ( 4.2) | 53 ( 3.2) |  |

# A-6 Details of covariate balance for crude, CBPS weighted and IV analysis

**Standardized difference > 0.1 (i.e. 1/10 of a standard deviation)*

|  | **Original Cohort** | | | |  |  | **CBPS Weighted** | | | |  |  | **DD as IV** | | | |  |  |
| --- | --- | --- | --- | --- | --- | --- | --- | --- | --- | --- | --- | --- | --- | --- | --- | --- | --- | --- |
| **Variable** | **X0.mean** | **X1.mean** | **X0.std.mean** | **X1.std.mean** | **Crude SMD** |  | **X0.mean.1** | **X1.mean.1** | **X0.std.mean.1** | **X1.std.mean.1** | **CBPS SMD** |  | **X0.mean** | **X1.mean** | **X0.std.mean** | **X1.std.mean** | **IV SMD** |  |
| age | 62.79 | 64.09 | 4.90 | 5.00 | 0.10 | * | 63.30 | 63.31 | 4.94 | 4.94 | 0.00 |  | 63.03 | 64.00 | 4.95 | 5.03 | 0.08 |  |
| sex2 | 0.80 | 0.75 | 1.94 | 1.83 | 0.11 | * | 0.78 | 0.78 | 1.89 | 1.88 | 0.01 |  | 0.77 | 0.77 | 1.85 | 1.84 | 0.01 |  |
| race2 | 0.92 | 0.92 | 3.44 | 3.42 | 0.01 |  | 0.92 | 0.92 | 3.43 | 3.42 | 0.01 |  | 0.94 | 0.92 | 3.64 | 3.56 | 0.08 |  |
| race3 | 0.03 | 0.03 | 0.17 | 0.19 | 0.02 |  | 0.03 | 0.03 | 0.18 | 0.19 | 0.01 |  | 0.02 | 0.03 | 0.11 | 0.20 | 0.09 |  |
| race4 | 0.00 | 0.00 | 0.01 | 0.09 | 0.08 |  | 0.00 | 0.00 | 0.03 | 0.04 | 0.01 |  | 0.00 | 0.00 | 0.07 | 0.02 | 0.05 |  |
| race5 | 0.03 | 0.02 | 0.19 | 0.14 | 0.05 |  | 0.03 | 0.03 | 0.17 | 0.17 | 0.00 |  | 0.02 | 0.03 | 0.13 | 0.20 | 0.07 |  |
| race6 | 0.01 | 0.02 | 0.09 | 0.15 | 0.06 |  | 0.01 | 0.01 | 0.11 | 0.12 | 0.01 |  | 0.01 | 0.01 | 0.11 | 0.10 | 0.01 |  |
| admYear2 | 0.06 | 0.11 | 0.22 | 0.41 | 0.20 | * | 0.08 | 0.08 | 0.30 | 0.30 | 0.00 |  | 0.09 | 0.06 | 0.33 | 0.23 | 0.10 |  |
| admYear3 | 0.06 | 0.12 | 0.21 | 0.45 | 0.24 | * | 0.08 | 0.08 | 0.29 | 0.30 | 0.01 |  | 0.08 | 0.07 | 0.29 | 0.28 | 0.01 |  |
| admYear4 | 0.06 | 0.10 | 0.24 | 0.39 | 0.15 | * | 0.08 | 0.08 | 0.29 | 0.30 | 0.01 |  | 0.09 | 0.06 | 0.36 | 0.21 | 0.15 | * |
| admYear5 | 0.08 | 0.10 | 0.28 | 0.34 | 0.06 |  | 0.08 | 0.08 | 0.30 | 0.30 | 0.00 |  | 0.12 | 0.05 | 0.42 | 0.18 | 0.24 | * |
| admYear6 | 0.11 | 0.08 | 0.36 | 0.28 | 0.08 |  | 0.10 | 0.10 | 0.33 | 0.33 | 0.00 |  | 0.11 | 0.08 | 0.38 | 0.28 | 0.10 |  |
| admYear7 | 0.11 | 0.08 | 0.36 | 0.28 | 0.09 |  | 0.10 | 0.10 | 0.33 | 0.33 | 0.00 |  | 0.06 | 0.13 | 0.20 | 0.46 | 0.25 | * |
| admYear8 | 0.13 | 0.09 | 0.41 | 0.29 | 0.12 | * | 0.11 | 0.11 | 0.36 | 0.35 | 0.01 |  | 0.08 | 0.15 | 0.25 | 0.47 | 0.21 | * |
| admYear9 | 0.13 | 0.10 | 0.40 | 0.30 | 0.10 | * | 0.12 | 0.11 | 0.37 | 0.36 | 0.01 |  | 0.12 | 0.12 | 0.37 | 0.36 | 0.01 |  |
| admYear10 | 0.14 | 0.10 | 0.42 | 0.30 | 0.12 | * | 0.13 | 0.13 | 0.38 | 0.38 | 0.00 |  | 0.15 | 0.11 | 0.46 | 0.31 | 0.14 | * |
| admYear11 | 0.12 | 0.09 | 0.39 | 0.29 | 0.10 | * | 0.11 | 0.11 | 0.35 | 0.35 | 0.00 |  | 0.09 | 0.16 | 0.27 | 0.48 | 0.21 | * |
| caria22 | 0.04 | 0.04 | 0.22 | 0.19 | 0.04 |  | 0.04 | 0.04 | 0.21 | 0.20 | 0.01 |  | 0.09 | 0.04 | 0.35 | 0.16 | 0.20 | * |
| caria23 | 0.03 | 0.02 | 0.17 | 0.11 | 0.06 |  | 0.02 | 0.02 | 0.15 | 0.16 | 0.00 |  | 0.03 | 0.01 | 0.19 | 0.09 | 0.11 | * |
| caria24 | 0.02 | 0.01 | 0.16 | 0.09 | 0.07 |  | 0.02 | 0.02 | 0.14 | 0.13 | 0.01 |  | 0.02 | 0.02 | 0.14 | 0.16 | 0.02 |  |
| IRSAD22 | 0.28 | 0.23 | 0.63 | 0.52 | 0.11 | * | 0.26 | 0.24 | 0.59 | 0.56 | 0.03 |  | 0.29 | 0.27 | 0.64 | 0.60 | 0.03 |  |
| IRSAD23 | 0.24 | 0.25 | 0.56 | 0.58 | 0.02 |  | 0.25 | 0.25 | 0.57 | 0.59 | 0.02 |  | 0.25 | 0.31 | 0.56 | 0.68 | 0.12 | * |
| IRSAD24 | 0.23 | 0.27 | 0.54 | 0.62 | 0.08 |  | 0.25 | 0.25 | 0.58 | 0.58 | 0.00 |  | 0.23 | 0.17 | 0.57 | 0.42 | 0.14 | * |
| IRSD22 | 0.26 | 0.23 | 0.60 | 0.53 | 0.07 |  | 0.25 | 0.24 | 0.58 | 0.56 | 0.02 |  | 0.30 | 0.32 | 0.65 | 0.69 | 0.04 |  |
| IRSD23 | 0.24 | 0.23 | 0.57 | 0.54 | 0.03 |  | 0.24 | 0.24 | 0.56 | 0.57 | 0.01 |  | 0.23 | 0.28 | 0.54 | 0.64 | 0.10 |  |
| IRSD24 | 0.24 | 0.26 | 0.56 | 0.61 | 0.05 |  | 0.25 | 0.25 | 0.58 | 0.59 | 0.00 |  | 0.24 | 0.17 | 0.60 | 0.41 | 0.19 | * |
| IER22 | 0.25 | 0.25 | 0.58 | 0.58 | 0.00 |  | 0.25 | 0.26 | 0.59 | 0.60 | 0.01 |  | 0.26 | 0.32 | 0.58 | 0.71 | 0.13 | * |
| IER23 | 0.27 | 0.26 | 0.61 | 0.58 | 0.03 |  | 0.27 | 0.26 | 0.60 | 0.60 | 0.01 |  | 0.34 | 0.31 | 0.72 | 0.67 | 0.05 |  |
| IER24 | 0.24 | 0.21 | 0.58 | 0.50 | 0.08 |  | 0.23 | 0.23 | 0.55 | 0.54 | 0.01 |  | 0.16 | 0.15 | 0.45 | 0.41 | 0.04 |  |
| IEO22 | 0.27 | 0.21 | 0.62 | 0.48 | 0.14 | * | 0.24 | 0.24 | 0.56 | 0.57 | 0.01 |  | 0.28 | 0.29 | 0.62 | 0.64 | 0.02 |  |
| IEO23 | 0.25 | 0.25 | 0.58 | 0.57 | 0.01 |  | 0.25 | 0.25 | 0.57 | 0.58 | 0.01 |  | 0.19 | 0.23 | 0.47 | 0.56 | 0.09 |  |
| IEO24 | 0.22 | 0.29 | 0.51 | 0.67 | 0.17 | * | 0.25 | 0.26 | 0.59 | 0.60 | 0.01 |  | 0.28 | 0.23 | 0.63 | 0.53 | 0.10 |  |
| adm.weekend2 | 0.25 | 0.34 | 0.55 | 0.75 | 0.20 | * | 0.28 | 0.28 | 0.62 | 0.63 | 0.01 |  | 0.26 | 0.30 | 0.57 | 0.68 | 0.10 |  |
| stemitm.offhours2 | 0.42 | 0.59 | 0.84 | 1.17 | 0.33 | * | 0.48 | 0.49 | 0.96 | 0.99 | 0.03 |  | 0.48 | 0.46 | 0.95 | 0.93 | 0.02 |  |
| o2dt | 148.12 | 164.60 | 0.85 | 0.94 | 0.09 |  | 154.95 | 156.88 | 0.89 | 0.90 | 0.01 |  | 155.86 | 149.47 | 0.92 | 0.88 | 0.04 |  |
| nyha2 | 0.58 | 0.55 | 1.16 | 1.10 | 0.06 |  | 0.56 | 0.56 | 1.14 | 1.13 | 0.01 |  | 0.50 | 0.54 | 1.00 | 1.08 | 0.08 |  |
| nyha3 | 0.11 | 0.13 | 0.34 | 0.40 | 0.05 |  | 0.11 | 0.12 | 0.36 | 0.37 | 0.01 |  | 0.10 | 0.10 | 0.33 | 0.33 | 0.00 |  |
| nyha4 | 0.05 | 0.06 | 0.21 | 0.28 | 0.06 |  | 0.05 | 0.05 | 0.24 | 0.24 | 0.00 |  | 0.05 | 0.05 | 0.21 | 0.24 | 0.03 |  |
| nyha5 | 0.21 | 0.20 | 0.52 | 0.50 | 0.02 |  | 0.21 | 0.21 | 0.51 | 0.51 | 0.00 |  | 0.29 | 0.27 | 0.65 | 0.61 | 0.04 |  |
| killip2 | 0.77 | 0.71 | 1.75 | 1.62 | 0.13 | * | 0.74 | 0.74 | 1.70 | 1.70 | 0.00 |  | 0.78 | 0.74 | 1.82 | 1.74 | 0.08 |  |
| killip3 | 0.11 | 0.13 | 0.35 | 0.39 | 0.05 |  | 0.12 | 0.12 | 0.37 | 0.37 | 0.00 |  | 0.11 | 0.12 | 0.33 | 0.39 | 0.05 |  |
| killip4 | 0.02 | 0.04 | 0.13 | 0.23 | 0.10 |  | 0.03 | 0.03 | 0.16 | 0.17 | 0.01 |  | 0.02 | 0.03 | 0.15 | 0.17 | 0.02 |  |
| killip5 | 0.08 | 0.10 | 0.27 | 0.35 | 0.08 |  | 0.08 | 0.08 | 0.29 | 0.30 | 0.00 |  | 0.07 | 0.08 | 0.26 | 0.29 | 0.03 |  |
| ohca1 | 0.10 | 0.12 | 0.31 | 0.40 | 0.09 |  | 0.10 | 0.11 | 0.33 | 0.35 | 0.01 |  | 0.09 | 0.10 | 0.31 | 0.35 | 0.05 |  |
| csind1 | 0.09 | 0.13 | 0.30 | 0.43 | 0.13 | * | 0.10 | 0.10 | 0.33 | 0.34 | 0.00 |  | 0.09 | 0.10 | 0.30 | 0.35 | 0.05 |  |
| sbp | 128.70 | 127.76 | 4.77 | 4.73 | 0.03 |  | 128.32 | 128.22 | 4.76 | 4.75 | 0.00 |  | 130.59 | 127.23 | 4.88 | 4.75 | 0.13 | * |
| dialysis1 | 0.00 | 0.00 | 0.06 | 0.07 | 0.01 |  | 0.00 | 0.00 | 0.07 | 0.07 | 0.00 |  | 0.00 | 0.00 | 0.06 | 0.05 | 0.01 |  |
| osa1 | 0.02 | 0.02 | 0.14 | 0.16 | 0.02 |  | 0.02 | 0.02 | 0.15 | 0.15 | 0.01 |  | 0.02 | 0.02 | 0.15 | 0.14 | 0.01 |  |
| rheum1 | 0.02 | 0.02 | 0.14 | 0.12 | 0.02 |  | 0.02 | 0.02 | 0.14 | 0.14 | 0.00 |  | 0.02 | 0.02 | 0.16 | 0.15 | 0.01 |  |
| cd1 | 0.04 | 0.06 | 0.20 | 0.27 | 0.07 |  | 0.05 | 0.05 | 0.22 | 0.22 | 0.00 |  | 0.04 | 0.05 | 0.21 | 0.24 | 0.03 |  |
| smokhist2 | 0.37 | 0.34 | 0.77 | 0.71 | 0.06 |  | 0.36 | 0.36 | 0.75 | 0.75 | 0.00 |  | 0.37 | 0.35 | 0.77 | 0.73 | 0.04 |  |
| smokhist3 | 0.28 | 0.28 | 0.63 | 0.62 | 0.01 |  | 0.28 | 0.28 | 0.62 | 0.62 | 0.00 |  | 0.28 | 0.28 | 0.62 | 0.62 | 0.00 |  |
| smokhist4 | 0.34 | 0.36 | 0.71 | 0.77 | 0.06 |  | 0.35 | 0.35 | 0.73 | 0.73 | 0.00 |  | 0.34 | 0.36 | 0.71 | 0.76 | 0.05 |  |
| dyslipid1 | 0.48 | 0.51 | 0.96 | 1.02 | 0.06 |  | 0.49 | 0.49 | 0.98 | 0.98 | 0.00 |  | 0.49 | 0.49 | 0.99 | 0.98 | 0.00 |  |
| asthma1 | 0.04 | 0.04 | 0.20 | 0.22 | 0.03 |  | 0.04 | 0.04 | 0.20 | 0.22 | 0.02 |  | 0.04 | 0.06 | 0.19 | 0.27 | 0.08 |  |
| copd1 | 0.04 | 0.05 | 0.18 | 0.26 | 0.09 |  | 0.04 | 0.04 | 0.21 | 0.21 | 0.00 |  | 0.04 | 0.05 | 0.19 | 0.23 | 0.04 |  |
| pvd1 | 0.03 | 0.04 | 0.15 | 0.23 | 0.08 |  | 0.03 | 0.03 | 0.18 | 0.19 | 0.01 |  | 0.03 | 0.03 | 0.16 | 0.20 | 0.04 |  |
| cadhist2 | 0.63 | 0.65 | 1.32 | 1.34 | 0.02 |  | 0.64 | 0.64 | 1.33 | 1.33 | 0.00 |  | 0.59 | 0.64 | 1.21 | 1.31 | 0.10 |  |
| cadhist3 | 0.33 | 0.32 | 0.71 | 0.69 | 0.02 |  | 0.33 | 0.33 | 0.69 | 0.70 | 0.00 |  | 0.36 | 0.32 | 0.76 | 0.68 | 0.08 |  |
| hpt1 | 0.52 | 0.55 | 1.04 | 1.11 | 0.07 |  | 0.53 | 0.54 | 1.06 | 1.07 | 0.01 |  | 0.53 | 0.56 | 1.06 | 1.12 | 0.06 |  |
| diab1 | 0.16 | 0.18 | 0.44 | 0.49 | 0.05 |  | 0.17 | 0.18 | 0.46 | 0.46 | 0.01 |  | 0.17 | 0.17 | 0.46 | 0.44 | 0.02 |  |
| diabdiet1 | 0.04 | 0.04 | 0.20 | 0.21 | 0.01 |  | 0.04 | 0.04 | 0.20 | 0.20 | 0.00 |  | 0.04 | 0.04 | 0.20 | 0.21 | 0.01 |  |
| diaboral1 | 0.10 | 0.11 | 0.33 | 0.36 | 0.03 |  | 0.11 | 0.11 | 0.35 | 0.35 | 0.00 |  | 0.10 | 0.09 | 0.34 | 0.32 | 0.03 |  |
| diabins1 | 0.03 | 0.04 | 0.18 | 0.22 | 0.04 |  | 0.04 | 0.04 | 0.19 | 0.20 | 0.01 |  | 0.04 | 0.04 | 0.21 | 0.21 | 0.00 |  |
| pmi1 | 0.11 | 0.15 | 0.34 | 0.45 | 0.11 | * | 0.12 | 0.13 | 0.37 | 0.39 | 0.01 |  | 0.12 | 0.14 | 0.36 | 0.41 | 0.05 |  |
| chf1 | 0.01 | 0.03 | 0.09 | 0.20 | 0.12 | * | 0.01 | 0.02 | 0.11 | 0.13 | 0.02 |  | 0.01 | 0.02 | 0.09 | 0.18 | 0.09 |  |
| prevpci1 | 0.10 | 0.12 | 0.32 | 0.39 | 0.07 |  | 0.10 | 0.11 | 0.34 | 0.35 | 0.01 |  | 0.10 | 0.12 | 0.33 | 0.37 | 0.04 |  |
| prevcabg | 0.02 | 0.03 | 0.12 | 0.20 | 0.09 |  | 0.02 | 0.02 | 0.15 | 0.15 | 0.00 |  | 0.03 | 0.02 | 0.18 | 0.13 | 0.05 |  |
| prevvalv1 | 0.00 | 0.01 | 0.04 | 0.10 | 0.06 |  | 0.00 | 0.00 | 0.07 | 0.07 | 0.00 |  | 0.00 | 0.00 | 0.04 | 0.07 | 0.03 |  |
| chfprior1 | 0.05 | 0.08 | 0.20 | 0.33 | 0.13 | * | 0.06 | 0.06 | 0.24 | 0.24 | 0.01 |  | 0.04 | 0.06 | 0.19 | 0.27 | 0.07 |  |
| bmi2 | 27.68 | 27.66 | 5.68 | 5.68 | 0.00 |  | 27.63 | 27.68 | 5.67 | 5.68 | 0.01 |  | 27.47 | 27.81 | 5.57 | 5.64 | 0.07 |  |
| timipre2 | 0.06 | 0.06 | 0.24 | 0.25 | 0.01 |  | 0.06 | 0.05 | 0.24 | 0.24 | 0.01 |  | 0.06 | 0.06 | 0.24 | 0.26 | 0.02 |  |
| timipre3 | 0.09 | 0.12 | 0.29 | 0.39 | 0.10 |  | 0.10 | 0.10 | 0.33 | 0.33 | 0.00 |  | 0.10 | 0.09 | 0.35 | 0.29 | 0.06 |  |
| timipre4 | 0.12 | 0.17 | 0.36 | 0.49 | 0.14 | * | 0.14 | 0.14 | 0.40 | 0.41 | 0.01 |  | 0.16 | 0.14 | 0.44 | 0.39 | 0.05 |  |
| lesiontype2 | 0.21 | 0.21 | 0.51 | 0.52 | 0.01 |  | 0.21 | 0.22 | 0.52 | 0.53 | 0.02 |  | 0.19 | 0.22 | 0.48 | 0.55 | 0.07 |  |
| lesiontype3 | 0.49 | 0.46 | 0.98 | 0.93 | 0.05 |  | 0.48 | 0.48 | 0.97 | 0.96 | 0.01 |  | 0.51 | 0.49 | 1.01 | 0.98 | 0.03 |  |
| lesiontype4 | 0.26 | 0.29 | 0.59 | 0.64 | 0.05 |  | 0.27 | 0.27 | 0.60 | 0.60 | 0.00 |  | 0.27 | 0.24 | 0.61 | 0.55 | 0.06 |  |
| prhasleftmain1 | 0.01 | 0.01 | 0.06 | 0.15 | 0.09 |  | 0.01 | 0.01 | 0.09 | 0.09 | 0.00 |  | 0.01 | 0.01 | 0.07 | 0.12 | 0.05 |  |
| prhasrightcoronary1 | 0.01 | 0.01 | 0.06 | 0.15 | 0.09 |  | 0.01 | 0.01 | 0.09 | 0.09 | 0.00 |  | 0.01 | 0.01 | 0.07 | 0.12 | 0.05 |  |
| prhaslad1 | 0.01 | 0.01 | 0.06 | 0.15 | 0.09 |  | 0.01 | 0.01 | 0.09 | 0.09 | 0.00 |  | 0.01 | 0.01 | 0.07 | 0.12 | 0.05 |  |
| prhasladp1 | 0.01 | 0.01 | 0.06 | 0.15 | 0.09 |  | 0.01 | 0.01 | 0.09 | 0.09 | 0.00 |  | 0.01 | 0.01 | 0.07 | 0.12 | 0.05 |  |
| prhascx | 0.09 | 0.13 | 0.30 | 0.42 | 0.12 | * | 0.11 | 0.11 | 0.34 | 0.35 | 0.01 |  | 0.10 | 0.12 | 0.32 | 0.37 | 0.05 |  |
| prhasdiagonal1 | 0.01 | 0.01 | 0.06 | 0.15 | 0.09 |  | 0.01 | 0.01 | 0.09 | 0.09 | 0.00 |  | 0.01 | 0.01 | 0.07 | 0.12 | 0.05 |  |
| prhasobtmarginal1 | 0.01 | 0.01 | 0.06 | 0.15 | 0.09 |  | 0.01 | 0.01 | 0.09 | 0.09 | 0.00 |  | 0.01 | 0.01 | 0.07 | 0.12 | 0.05 |  |
| prhassvg1 | 0.01 | 0.01 | 0.06 | 0.15 | 0.09 |  | 0.01 | 0.01 | 0.09 | 0.09 | 0.00 |  | 0.01 | 0.01 | 0.07 | 0.12 | 0.05 |  |
| prhasbypassg1 | 0.01 | 0.01 | 0.06 | 0.15 | 0.09 |  | 0.01 | 0.01 | 0.09 | 0.09 | 0.00 |  | 0.01 | 0.01 | 0.07 | 0.12 | 0.05 |  |
| prhasnoreflowr1 | 0.01 | 0.01 | 0.06 | 0.15 | 0.09 |  | 0.01 | 0.01 | 0.09 | 0.09 | 0.00 |  | 0.01 | 0.01 | 0.07 | 0.12 | 0.05 |  |
| prhaslongstent1 | 0.01 | 0.01 | 0.06 | 0.15 | 0.09 |  | 0.01 | 0.01 | 0.09 | 0.09 | 0.00 |  | 0.01 | 0.01 | 0.07 | 0.12 | 0.05 |  |
| prhasrefvess2_51 | 0.01 | 0.01 | 0.06 | 0.15 | 0.09 |  | 0.01 | 0.01 | 0.09 | 0.09 | 0.00 |  | 0.01 | 0.01 | 0.07 | 0.12 | 0.05 |  |
| prhasbifurc1 | 0.01 | 0.01 | 0.06 | 0.15 | 0.09 |  | 0.01 | 0.01 | 0.09 | 0.09 | 0.00 |  | 0.01 | 0.01 | 0.07 | 0.12 | 0.05 |  |
| prhascto1 | 0.01 | 0.01 | 0.06 | 0.15 | 0.09 |  | 0.01 | 0.01 | 0.09 | 0.09 | 0.00 |  | 0.01 | 0.01 | 0.07 | 0.12 | 0.05 |  |
| procstentcount1 | 0.01 | 0.01 | 0.06 | 0.15 | 0.09 |  | 0.01 | 0.01 | 0.09 | 0.09 | 0.00 |  | 0.01 | 0.01 | 0.07 | 0.12 | 0.05 |  |

#
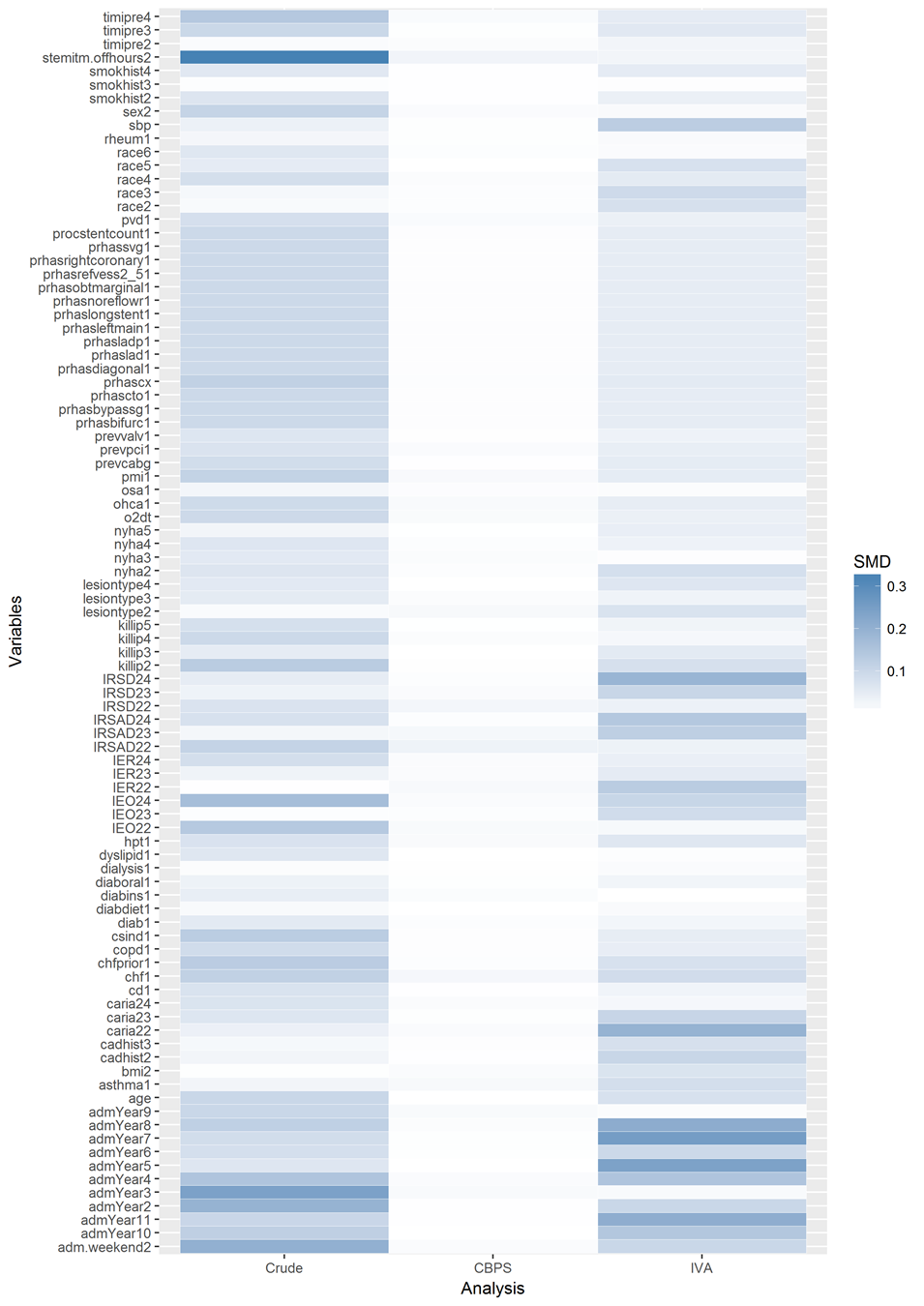
A-7 Visualization of covariate balance for crude, CBPS weighted and IV analysis
